# Closing the communication gap in neonatal inter-hospital transfer: a neonatal referral form for resource-limited settings - a modified e-Delphi-consensus study

**DOI:** 10.12688/f1000research.50980.2

**Published:** 2022-06-07

**Authors:** Oscar Mwizerwa, Christian Umuhoza, Mark H. Corden, Tom Lissauer, Peter Thomas Cartledge

**Affiliations:** 1 Department of Pediatrics and Child Health, University of Rwanda, Kigali, Rwanda; 2 Department of Pediatrics, University Teaching Hospital of Kigali, Kigali, Rwanda; 3 Rwanda Human Resources for Health (HRH) Programme, Ministry of Health, Kigali, Rwanda; 4 Division of Hospital Medicine, Department of Pediatrics, Children’s Hospital Los Angeles, Los Angeles, California, USA; 5 Department of Neonatology, Imperial College Healthcare Trust, London, UK; 6 Royal College of Paediatrics and Child Health (RCPCH UK), Kigali, Rwanda; 7 Department of Pediatrics, Yale University, New Haven, Connecticut, USA; 8 Centre for Paediatrics and Child Health, Imperial College London, London, UK

**Keywords:** Infant, Newborn, Referral and consultation, Patient transfer, Communication, Developing countries, Rwanda

## Abstract

**Background:** Standardised neonatal referral forms (NRFs) facilitate effective communication between healthcare providers and ensure continuity of care between facilities, which are essential for patient safety. We sought to determine the essential data items, or core clinical information (CCI), that should be conveyed for neonatal inter-hospital transfer in resource-limited settings (Rounds 1 to 3) and to create an NRF suitable for our setting (Round 4).

**Methods:** We conducted an international, four-round, modified Delphi-consensus study. Round-1 was a literature and internet search to identify existing NRFs. In Round-2 and -3, participants were Rwandan clinicians and international paediatric healthcare practitioners who had worked in Rwanda in the five years before the study. These participants evaluated the draft items and proposed additional items to be included in an NRF. Round-4 focused on creating the NRF and used five focus groups of Rwandan general practitioners at district hospitals.

**Results:** We identified 16 pre-existing NRFs containing 125 individual items. Of these, 91 items met the pre-defined consensus criteria for inclusion in Round-2. Only 33 items were present in more than 50% of the 16 NRFs, confirming the need for this consensus study. In Round-2, participants proposed 12 new items, six of which met the pre-defined consensus criteria. In Round-3, participants scored items for importance, and 57 items met the final consensus criteria. In Round-4, 29 general practitioners took part in five focus groups; a total of 16 modifications were utilised to finalise the NRF.

**Conclusions:** We generated a novel, robust, NRF that may be readily employed in resource-limited settings to communicate the essential clinical information to accompany a neonate requiring inter-hospital transfer.

## Introduction

Globally, neonatal mortality fell to 19 per 1000 live births in 2015 with the aim to reduce neonatal mortality to less than 12 per 1000 live births by 2030. ^1^ In Rwanda, the neonatal mortality rate has fallen from 37 to 19 per 1000 live births over 15 years (2005-2020). ^2, 3^ The reason for this decline is multifactorial, but the centralisation of specialist care has contributed to an increase in the number of neonatal transfers between low-level units and more specialised hospitals. Healthcare systems should be redesigned to deliver care that is safe, effective and efficient, whilst meeting the multiple needs of patients, including neonates. ^4^

Transport of neonates carries a range of risks, and adverse outcomes in up to 40% of transported neonates have been reported, with higher mortality associated with long-distance and duration of transport. ^5– 7^

A specialised transport system for neonates can mitigate against these risks. ^8^ In most resource-limited settings, such systems do not yet exist, and for many settings are currently out of reach. ^9^ For example, in Rwanda, a general patient-referral transport system is in place, but specialised neonatal transfer is lacking. ^10^ To optimise the transfer of care when transferring a newborn, it is paramount that the essential demographic and clinical information is shared between the referring and receiving sites. Many sick neonates in Rwanda are transferred to care facilities with inadequate clinical information to allow optimal care of the child. We questioned why health-professionals are not sharing this information adequately. Human factors are central to this and adapting the referral process was required.

Neonatal referral forms (NRFs) are standardised instruments that aid in ensuring high-quality handover of medical history, which include demographics, pre-transfer care, and, potentially, the transport course of the neonate. ^11– 19^ The quality of the shared clinical information has the potential to improve the outcome for the neonate and also reduce the repetition of investigations and treatments, thus decreasing the cost to families and facilities. ^18^ In the resource-limited setting, we identified that there is a lack of consensus about which clinical information should be communicated between hospital sites, and therefore included in a setting appropriate NRF. ^20^

### Objectives

We sought to determine consensus on the essential data items (core clinical information, CCI) that should be conveyed for neonatal inter-hospital transfer in a resource-limited setting, and to create an NRF suitable for our setting. The aimed output was for an NRF suitable for “day-to-day” clinical use, in order to improve quality of care, rather than for future research data-collection.

## Methods

### Study design

We employed a four-round, modified Delphi technique to identify the CCI that would be essential to communicate during neonatal transfer. Reporting of the study is in accordance with the Sinha and Williamson checklists for creating a “Core Outcome Set” using Delphi techniques. ^21, 22^ The Delphi methodology was chosen as a consensus tool as it allows large numbers of individuals across diverse locations and areas of expertise to be included anonymously, thus avoiding the domination of the consensus process by one or a few experts. It also can be undertaken remotely, removing the need for participants to travel. We modified the Delphi technique in two respects. In Rounds-1 to -3, we sought to meet the first objective of the study, namely to gain consensus on the CCI that should be conveyed for neonatal inter-hospital transfer in a resource-limited setting. In Round-4, we sought to meet the second objective to create an NRF suitable for our setting and employed focus-groups of clinicians who work in the setting to gain consensus.

### Round-1

A comprehensive literature and internet search was undertaken to identify pre-existing NRFs and research articles describing NRFs and/or CCI required for inter-hospital transfer of a neonate in the resource-limited setting. In Round-1 we used a Medline/PubMed literature search (rather than participants) in order to help improve response rates in the subsequent rounds, as well as to provide an initial sound CCI set to participants. ^23^ The search strings included MeSH terms and synonyms for “neonates”, “transportation of patients,” and “resource-limited settings” ( Supplementary Table 1, *Extended data* ^36^). We search for grey literature using both an internet search and using informal networking of contacts known to the authors, we then contacted local (Rwandan) healthcare facilities as well as experienced paediatricians in the region (Malawi, Uganda, and Kenya), by email, to identify any NRFs relevant to our setting, which were not published. We aimed for a minimum of 10 NRFs. Due to the low number of NRFs identified from resource-limited settings, the Medline/PubMed search was then repeated for NRFs, and articles containing NRFs, from outside of the resource-limited setting ( Supplementary Table 1, *Extended data* ^36^), by removing the “developing countries” search string. The individual items found in each NRF were then coded by two authors (OM and PC). During the coding process, items were intentionally removed if they were judged not to be relevant to resource-limited settings where there is no dedicated transport team (e.g., therapeutic hypothermia). Consensus in this round was pre-defined as any item that was used in two or more of the identified NRFs. These items were then used to create the first draft of our CCI list in preparation for Rounds-2 and -3 of the Delphi process.

### Selection of participants for Round-2 and -3

Participants from four groups of clinicians were eligible to be included: (i) Rwandan clinicians working in clinical paediatric practice, including all paediatric specialists and senior paediatric residents practicing in Rwanda, identified via the Rwanda paediatric email group; (ii) members of the Rwandan Neonatal Working Group (NWG) including paediatricians, public health specialists and policymakers, identified through the chair of the NWG; (iii) general practitioners (GPs; clinicians working in Rwandan district hospitals) identified through the NWG; (iv) non-Rwandan paediatricians with paediatric clinical experience in Rwanda through the Human Resources for Health (HRH) project identified from the Ministry of Health (MoH) database of HRH faculty. ^24^

### Round-2 (open-ended questionnaire)

In Round-2, the draft CCI list from Round-1 was divided into eight themes/sections. Participants were informed about the process involved in gaining the first draft CCI in Round-1. Each section contained a list of the included items in the first draft of the CCI. The list of items was then presented to participants with an open question using a “free text” option at the end of each section.

Participants were presented with a scenario: “We want you to imagine that you are either transferring or receiving a sick neonate who is being transferred between facilities in a resource-limited setting (e.g. Rwanda).” They were then asked what additional clinical items they would add to that section/theme. Consensus was pre-defined as any additional item suggested independently by two or more participants; these were then added to the second draft CCI for Round-3. Non-participation in Round-2 did not exclude participation in Round-3, but no additional participants were invited to Round-3.

### Round-3 (closed questions)

All items from the second draft of the CCI were listed in their themes/sections. Each item was presented with feedback from Round-1 in the form of the percentage of articles/NRFs that contained the item, or if it was a new addition from Round-2. After piloting the Round-3 Delphi questionnaire, several items that described similar clinical information were combined to minimise bias from questionnaire fatigue (e.g., stimulation, bag-mask ventilation, etc. were combined into “resuscitation”) ( Table 1). The participants were provided with the same clinical scenario as in Round-2 and were then asked to rank the importance of each CCI item on a nine-point Likert scale. Consensus for inclusion in the final CCI was pre-defined as greater than 70% of participants scoring 7–9 (important) AND less than 15% of participants scoring 1-3 (not important) as per GRADE/COMET criteria. ^25, 26^ Participants were informed of the pre-defined consensus to engage them in the process. Participants gave their year of birth and initials in Round-2 and -3 to assess attrition rate.

**Table 1.** Core clinical information items and domains for Rounds 1 to 3.

		Round-1		Round-2		Round-3	
**CCI domain/section**	Total number of items described in at least 1 NRF	Number of items found only in 1 NRF	Number of items for first draft of CCI (described in at least ≥2 NRFs (included)	New items from participants meeting consensus	Items merged with items to minimise questionnaire fatigue	Total number of items presented in Round-3	Final number of items for the CCI list
Hospital details (Introduction)	16	6	10	0	1	9	8
Patient identification	13	4	9	2	0	11	8
Clinical history at referral	6	3	3	1	0	4	4
Maternal medical and antenatal history	17	4	13	6	0	19	9
Labor details	14	4	11	2	0	13	11
Neonatal past medical history	19	1	17	1	13	5	4
Management at the referring hospital	20	1	19	0	7	12	11
Miscellaneous	10	1	9	0	0	9	2
**Totals**	**115**	**24**	**91**	**12**	**21**	**82**	**57**

CCI, core clinical information; NRF, neonatal referral form.

### Round-2 and Round-3 data collection and analysis

The Round-2 and Round-3 questionnaires were hosted and completed using Google Forms ^®^ and distributed to participants via email (see *Extended data* for Round-2 and Round-3 questionnaires ^36^). Participants were given two weeks to answer each questionnaire from Round-2 and Round-3 with email reminders sent after one week. We aimed for a minimum of 15 respondents in each round. ^27^ Google Forms ^®^ provides data in a downloadable Microsoft Excel ^®^ spreadsheet (V16 for Macintosh) which was used to code and describe statistics (i.e., median, mean, standard deviation, attrition rate) where appropriate.

### Round-4 - creating the final NRF

Rounds 1-3 provided consensus from the participants on the CCI that should be conveyed for neonatal inter-hospital transfer in a resource-limited setting and met the first objective of the study. The second objective of the study sought to use this CCI list to create an NRF for clinical use. The CCI items were therefore employed to create a draft NRF. This draft NRF was produced on two pages to enable double-sided printing on a single sheet of A4 paper to keep costs low and facilitate use. The draft NRF was then reviewed at a face-to-face meeting of the Rwandan MoH Neonatal Technical Working Group (NTWG). At this point, amendments were made to the structure and format of the NRF. The members of the NTWG then agreed that a fourth round of the Delphi process was required to ensure that the NRF was ready to be utilised in the clinical setting.

*Eligible participants for Round-4 (focus groups)*

In Rwanda, and many similar settings, referrals between the district and regional/tertiary hospitals are usually made by General Practitioners (GPs). GPs are medically qualified doctors who have not undertaken specialist training. Eligible participants for the Round-4 focus groups, were, therefore, GPs who had referred neonates to regional and tertiary hospitals. Focus group participants were selected opportunistically as those GPs that were available, and consenting, at the hospital sites on the dates the Principle Investigator visited each of the five hospitals.

*Focus groups*

Focus groups were undertaken in a quiet room, with no patients or other professionals present. The participants were given hard paper copies of the NRF and an explanation of the purpose of the process and the objective of producing an NRF for clinical use in Rwanda. They were then asked to review the NRF and collectively make suggestions to improve and amend the NRF. No interview guide was used. Focus groups were undertaken in a mix of Kinyarwanda, English and French (official national languages of Rwanda). Recordings were not taken, with the Principal Investigator (OM) taking field notes.

*Coding and analysis*

Field notes were coded by two coders (OM and PC) using Microsoft Excel, and consensus identified if a code was introduced in two or more separate focus groups. These themes were used to amend the NRF further.

*Approval*

Finally, the NRF was re-reviewed by the MoH NTWG and the MoH National Health Referral System committee, with no further amendments to the content of the form made.

### Ethics statement

*Ethics approval*

The study protocol was reviewed and approved by the University of Rwanda College of Medicine and Health Sciences Institutional Review Board (IRB) (Ref: 002/CMHS IRB/2018). There were no significant physical, emotional, social, financial or legal risks to participants identified.

*Consent:*

For Rounds-2 and -3 information regarding the study was provided to participants at the same time as the online google questionnaire and completion of the questionnaires implied informed consent of participation. For Round-4 verbal information was given to participants and participation was deemed as implied consent.

*Confidentiality*

The questionnaire was fully anonymised, and email invitations were sent individually to maintain confidentiality. Participant demographics and response data were obtained via Google forms ^®^, which is password-protected and accessed only by the principal investigator and both supervisors of this project.

## Results

### Round-1

We identified a total of 16 NRFs. Initial searches related to resource-limited settings identified two NRFs from Rwandan Provincial hospitals (Rwamagana and Ruhengeri). Fourteen NRFs were identified from upper-middle and high-income countries: eight from the United Kingdom, five from the United States and one from South Africa. These 16 NRFs contained a total of 125 individual items, of which ten items were immediately removed as being not relevant to the CCI in a resource-limited setting (e.g., therapeutic hypothermia). Ninety-one of the remaining 115 items (79%) met the pre-defined criteria for consensus to be included in the first draft of the CCI. These items were listed individually, grouped under eight relevant sections to aid interpretation by participants ( Table 1). Each NRF contained a mean of 34 items (min = 11, max = 52). Only 33 of the 91 identified items (36%) were present in more than half of the 16 NRFs, confirming the need for this consensus study.

### Response rate

124 participants were contacted. The response rate was 32 (25%) and 33 (27%) participants for Round-2 and Round-3, respectively. This response rate exceeded our desired sample size of 15, required for consensus, in each round. Eleven of the 32 (33%) participants from Round-2 also completed Round-3; thus, 22 of 33 (67%) participants in Round-3 were new responders. Participants in Round-2 and -3 had a mean of 14 years and 13 years of paediatric experience, respectively ( Table 2).

**Table 2.** Baseline characteristics of participants of Round-2 and Round-3 of Delphi process.

		Round-2 (n=33)	Round-3 (n=34)
**Age (years)**	Mean (SD)	42.2 (±12.5)	40.9 (±10.7)
**Level of expertise**	General Pediatrician	11 (34%)	18 (55%)
	Senior Resident	13 (41%)	8 (24%)
	Pediatric subspecialist (non-neonatologist)	5 (16%)	3 (9%)
	Neonatologist	1 (3%)	3 (9%)
	Other	2 (6%)	1 (3%)
**Location**	East Africa	22 (69%)	20 (61%)
	USA	8 (25%)	12 (36%)
	Other	2 (6%)	1 (3%)
**Primary institution**	Referral Hospital	27 (84%)	23 (70%)
	District Hospital	2 (6%)	7 (21%)
	Others	3 (9%)	3 (9%)
**Employment status**	Full-time	25 (78%)	29 (88%)
	Part-time	4 (13%)	3 (9%)
	Retired	3 (9%)	1 (3%)
**Country of medical degree**	Rwanda	18 (56%)	16 (49%)
	USA	9 (28%)	11 (33%)
	Other	5 (15%)	6 (18%)
**Experience post-graduation from medical school**	Mean (SD)	14.1 (±13.1)	12.9 (±10.5)

### Round-2

All sections/themes had additional items suggested by participants, with 20 participants suggesting new additions to the “Patient Identification” section. Fifty-two items were suggested that were already present in the Round-1 CCI and were therefore excluded. Thirty-three new items were suggested. Twelve (36%) of these were independently suggested by two or more participants and therefore met the pre-defined consensus criteria and were added to the existing 91 items from Round-1 to form the draft CCI list of 103 items for Round-3 ( Table 1).

**Figure 1.**
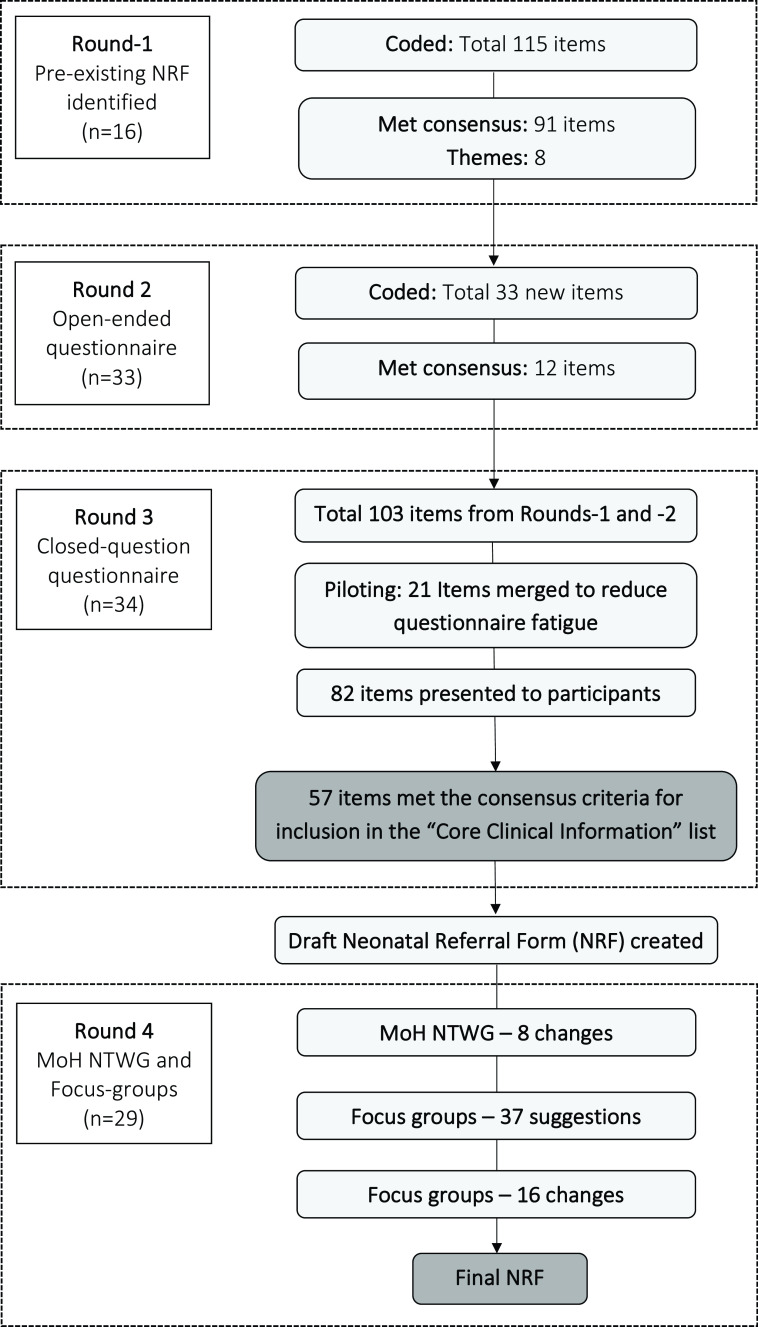
Delphi flow diagram. MoH NTWG, Ministry of Health Neonatal Technical Working Group; NRF, neonatal referral form.

Piloting of the Delphi questionnaire between Round-2 and Round-3 revealed that 21 items could be merged with existing items ( Figure 1). For example, in Round-2, seven different types of birth resuscitation were described (e.g., bag-valve-mask, stimulation, etc.); these were merged with the item of “resuscitation at birth.” This was to reduce questionnaire fatigue, which was reported by the piloting participants. Therefore, after merging these items, the list reduced from 103 to 82 items.

### Round-3

The questionnaire was again divided into the same eight sections/domains, to form a second draft CCI list. Eighty-two items were presented individually for scoring of “importance” in Round-3 ( Supplementary Table 2, *Extended data* ^36^). Of these, 57 items (70%) met the pre-defined consensus criteria to be included in the final CCI list ( Supplementary Table 3, *Extended data* ^36^). Seven of the 12 items (58%) suggested in Round-2 met the inclusion criteria for the final CCI list. This list was then used to generate the first draft of the NRF (Supplementary File 3, *Extended data* ^36^).

### Round-4 (focus groups)

The draft NRF was then reviewed at the Rwandan MoH Neonatal Technical Working Group (NTWG), and eight amendments were made ( Supplementary Table 4, *Extended data* ^36^). Twenty-nine general practitioners then took part in five focus groups to review the NRF, with each focus group lasting between 60 and 90 minutes. The five focus groups were undertaken at three rural district hospitals [Gahini (n = 5), Nemba (n = 9) and Ruli (n = 4)] and two urban provincial (secondary) hospitals [Ruhengeri (n = 8), Rwamagana (n = 3)]. The focus-groups created a total of 37 new suggestions for modifying the NRF ( Supplementary Table 4, *Extended data* ^36^). Nineteen of these suggestions were made in two or more of the focus groups. Sixteen of these changes were incorporated into the final NRF (Supplementary File 4, *Extended data* ^36^); three were not feasible, namely, creating a French version, adapting a version specific for health centres and reducing the size of the NRF.

## Discussion

We sought to determine the essential data items that should be conveyed for neonatal inter-hospital transfer in a resource-limited setting. By employing Delphi techniques, we have generated consensus on what information should be included in a neonatal referral form in order to close the communication gap in inter-hospital transfer in Rwanda. Additionally, given the breadth of experience of our participants, we anticipate that the form is readily adaptable to other similar resource-limited settings.

Precise records are an essential component of neonatal inter-hospital transfer. ^28^ However, there is limited evidence for how such data should be communicated. For example, the NRFs we identified in Round-1 were heterogeneous in nature, with only 33 (36%) of the 91 items identified being present in more than half of the 16 NRFs. Therefore, many NRFs may be excluding important data points because robust methods were not employed to develop them. We found that these existing NRFs contained a mean of 34 items, which is less than the 57 items we identified in Rounds -1 to -3 and therefore these NRFs may not be comprehensive. Items in the NRF that are unique to resource-limited settings include modes of transport (e.g. motorcycle, walking), conditions in pregnancy (e.g. tuberculosis), and perinatal infant care (e.g. tetracycline eye ointment). Despite having more items, our NRF fits on two pages as practicalities such as printing costs are important in our setting. The practicality of ensuring that the NRF could be printed on a single A4 double-sided sheet of paper, had resulted in the use of a small font size, which may be challenging for persons with reduced eye-sight, and that the ergonomics of the form may not be optimal.

Communication errors have been established as a potential cause of significant adverse events during transport. ^29^ By encouraging strict adherence to data collection and sharing, NRFs can also assist with providing a standardised patient handoff, which has been identified as a quality metric for neonatal transport. ^30, 31^ Also, NRFs allow for tracking data to assist with clinical benchmarking for transport outcomes, a much-needed measure in neonatal inter-hospital transfer and neonatal registries. ^32– 34^

By utilising a modified Delphi consensus process, we have incorporated core items that previous NRFs included while tailoring the form for use specifically in a resource-limited setting. The participants contributing to the consensus process had a broad length of experience and diverse backgrounds and training. Their broad expertise helped contribute extensive and informed feedback in Round-2 and -3 of the Delphi. Given that two-thirds of these participants were from East African countries, we feel that the consensus process produced CCI items that are generalisable to other resource-limited settings. Using focus groups of general practitioners in Round-4 of the study has made the NRF relevant and practical to the professionals working in the health facilities that are required to transfer neonates for more specialist care.

### Study limitations

We identified several possible limitations to our study. First, only two of the 16 NRFs obtained in Round-1 were from resource-limited settings. The content validity of the 14 other NRFs may, therefore, be decreased for our setting. There may be other NRFs from a similar setting, but despite internet/literature searches and contacting local networks, these were not identifiable. However, we feel that this is minimised by the participant contributions in Rounds-2, -3 and -4. Second, the participants involved were all physicians. Parents and nursing staff were not included. It was felt that parents were unlikely to understand the terminology or the nature of the clinical information being presented. However, adding nursing staff could have affected our results by eliminating certain items or introducing additional novel items in our NRF. Only four board-certified neonatologists participated in our study. In practice, many of the general paediatricians who participated in our study, particularly from resource-limited settings, care primarily for neonates but simply do not have official certification as subspecialists. Hence, we feel that the expertise of our participants remains highly relevant to the setting and scope of our study. Regarding potential biases, there are 91 items in the first draft of the CCI list. Asking participants to score the importance of all of these items could result in questionnaire fatigue and bias the results of the later outcomes. To mitigate against this possibility, the questionnaire was split into eight themes/sections, and some items were combined in the final round.

### Clinical application

Despite these limitations, we anticipate that our NRF can be used in its current form at any centre that refers neonates to institutions that provide a higher level of care in resource-limited settings. If employed in other settings, the language and terminology would need to be modified where appropriate and the items assessed for local suitability.

The aimed output was for an NRF suitable for “day-to-day” clinical use, in order to improve quality of care, rather than for future research data-collection. The NRF is now in national clinical use in Rwanda. ^35^ It was not initially field-tested in its double-sided, A4 format but has now been adapted by the Ministry of Health to fit onto a double-spread A3 sized pad of carbon-copied paper so that the completed form can be sent to the receiving hospital, with the sending facility keeping a copy. This is used at all levels of referral by different professionals. Standard practice is that doctors complete the form in hospitals and nurses and/or midwives complete it at health centre level. There is currently no formal training in its use.

### Future research

Further assessment will be required to assess how frequently the form is used, how accurately it is completed and if it improves outcomes. The design/maintenance and use of the form is now undertaken by the Neonatal Working Group of the Ministry of Health, which includes multi-disciplinary professionals, who are best placed to evaluate its ongoing implementation. This NRF represents an important first step in standardising data collection in our setting in, and may be helpful in other resource-limited settings. Though the form is designed for day-to-day clinical use, the data generated has be potential to form the foundation for future quality improvement initiatives in neonatal inter-hospital transfers or centres establishing neonatal registries. ^34^

## Conclusion

Adverse outcomes occur in up to 40% of neonates transported between hospitals, and there are currently no standardised, robustly designed, communication tools for the transport of neonates in resource-limited settings. We have used Delphi consensus methods to generate a novel NRF to close the communication gap in inter-hospital transfer in the resource-limited setting, such as Rwanda, where it is now in national clinical use. The NRF is freely available on Harvard Dataverse ^36^ with a CC0 licence, allowing other settings to use and adapt the NRF to their own setting needs.

## Data availability

### Underlying data

Harvard Dataverse: A Neonatal Referral Form For Resource-Limited Settings - A Modified Delphi-Consensus Study. https://doi.org/10.7910/DVN/Q0ZGDZ. ^36^

This project contains the following underlying data:

- Round-1 - NRF e-Delphi.tab (Round-1 dataset)
- Round-2 - NRF e-Delphi.xlsx (Round-2 dataset)
- Round-3 - NRF e-Delphi.tab (Round-3 dataset)
- Round-4 - NRF e-Delphi.tab (Round-4 dataset)

### Extended data

Harvard Dataverse: A Neonatal Referral Form For Resource-Limited Settings - A Modified Delphi-Consensus Study. https://doi.org/10.7910/DVN/Q0ZGDZ. ^36^

This project contains the following extended data:

- e-Delphi consent form.docx (Consent description for Round-2 and Round-3)
- Google Forms - Questionnaire for Round-2.pdf (Round 2 Questionnaire)
- Google Forms - Questionnaire for Round-3.pdf (Round 3 Questionnaire)
- Supplementary_File_3_RaNRF_Round3.docx (Draft neonatal referral form generated in Round-3)
- Supplementary_File_2_FINAL_NRF_after_Round_4.docx (Final neonatal referral form for resource-limited settings - for public use)
- Supplementary_Table_1_Search_terms.docx (Search terms used in Round-1)
- Supplementary_Table_2_Round_3.docx (Round-3 responses and consensus application)
- Supplementary_Table_3_Final_CCI_list.docx (Final CCI list at end of Round-3)
- Supplementary_Table_4_Round_4_coding.docx (Coding used in Round-4)

Data are available under the terms of the Creative Commons Zero “No rights reserved” data waiver (CC0 1.0 Public domain dedication).
