# Supplementary material for: Closing the communication gap in neonatal inter-hospital transfer: a neonatal referral form for resource-limited settings - a modified e-Delphi-consensus study
Source: F1000Res. 2022 Jun 7;10:365. Originally published 2021 May 10. [Version 2] doi: 10.12688/f1000research.50980.2 (PMC9201411; doi:10.12688/f1000research.50980.2)
Supplement: Peer review history

# Peer review: Reviewer response for version 2

**DOI:** 10.5256/f1000research.125455.r139935

**Published:** 2022-06-15

**Authors:** Peter Lachman

**Affiliations:** 1 Lead Faculty Quality Improvement Programme, Royal College of Physicians of Ireland, Dublin, Ireland

The authors have addressed the issue of clinical implementation. The study is well constructed and the value of the work can now be assessed going forward:

- is the form being used reliably?
- has it been used as intended?
- does it need modification?
- is there better communication?
- are the neonatal transfers safer?
- have clinical outcomes for neonates e.g. morbidity and mortality improved?

I look forward to seeing the next stage of this important work

Is the work clearly and accurately presented and does it cite the current literature?

Yes

If applicable, is the statistical analysis and its interpretation appropriate?

Yes

Are all the source data underlying the results available to ensure full reproducibility?

Yes

Is the study design appropriate and is the work technically sound?

Yes

Are the conclusions drawn adequately supported by the results?

Yes

Are sufficient details of methods and analysis provided to allow replication by others?

Yes

Reviewer Expertise:

Patient Safety, Quality Improvement and implementation, Paediatrics

I confirm that I have read this submission and believe that I have an appropriate level of expertise to confirm that it is of an acceptable scientific standard.

# Peer review: Reviewer response for version 1

**DOI:** 10.5256/f1000research.54082.r125910

**Published:** 2022-03-28

**Authors:** Peter Lachman

**Affiliations:** 1 Lead Faculty Quality Improvement Programme, Royal College of Physicians of Ireland, Dublin, Ireland

This paper provides a report on the development of a neonatal referral form for use in a low resource setting. This is an important development to improve reliability of communication thereby improving reliability of care and neonatal outcomes.

- The rationale for the study is well explained i.e. the lack of a standardized referral process in the context of high neonatal mortality and the need for improved communication on referral.
- The context is relevant and important.
- The rationale for the study is clear.
- The study methodology is described well.
- The literature search for similar tools appears to be good – though there may be grey literature i.e unpublished referral forms not available for review.
- The modified Delphi appears to be well run and addresses possible bias.
- The results are described in sufficient detail.
- The supplementary data provides the important background to the paper as well as the final version of the form.

As a paper reporting the development of a tool to improve the reliability of information transfer, the authors have met the aim of the study i.e. developing a form co-designed by referrers and receivers of the referral using a Delphi approach.

 I would have expected the theory of standardization of communication to be discussed in the context of patient safety theory, in particular human factors and reliability .

- The layout of the form could also be designed more ergonomically.
- Was the layout tested in the field to see how best to set out the required  information?

The paper does not address the following though the authors note that the form “i *s now in national clinical use in Rwanda"*

- Does the form work i.e. do clinicians use it reliably where reliability is defined as completion of  all required information accurately 100% of the time?
- Are any modifications required especially in design of form?
- Are all the items necessary in real life outside the research environment where it was developed.
- What training is required to have the form used? Is there a training manual?
- Who fills out the form – nurses or doctors?
- On case by case basis has the form improved the outcome for babies when filled out correctly?

I note that that these questions are for future research. However a comment on this and on how the implementation of the form is carried out and scaled up would be a good follow up study. A neonatal collaborative across the country may be the way to ensure the form is used on a reliable basis.

 A note on the above would add value to the findings of the paper.

Is the work clearly and accurately presented and does it cite the current literature?

Yes

If applicable, is the statistical analysis and its interpretation appropriate?

Yes

Are all the source data underlying the results available to ensure full reproducibility?

Yes

Is the study design appropriate and is the work technically sound?

Yes

Are the conclusions drawn adequately supported by the results?

Yes

Are sufficient details of methods and analysis provided to allow replication by others?

Yes

Reviewer Expertise:

Patient Safety, Quality Improvement and implementation, Paediatrics

I confirm that I have read this submission and believe that I have an appropriate level of expertise to confirm that it is of an acceptable scientific standard, however I have significant reservations, as outlined above.

# Response

**Published:** 2022-05-20

**Authors:** Peter Cartledge

**Affiliations:** Yale University (USA) Human Resources for Health (Rwanda), Rwanda

We would like to thank the reviewer for their comments and input into the article, which we feel contribute to the article. Here we respond to each comment, point by point:

- *The literature search for similar tools appears to be good – though there may be grey literature i.e unpublished referral forms not available for review*
- **Response**: This is a very valid point. During the study design phase, we did undertake a “grey search” using both an internet search and through networking of contacts known to us. We have amended the text in “Methods” > ”Round-1” to hopefully make this a bit more clear to readers.
- *I would have expected the theory of standardization of communication to be discussed in the context of patient safety theory, in particular human factors and reliability.*
- **Response**: We have added some new text to the background putting the study in context of human factors. We can see that much of what we did employed Human Factors/Ergonomics (HFE) theory, but we feel it would be disingenuous to imply that we used this approach formally in our methodology.
- *The layout of the form could also be designed more ergonomically.*
- **Response**: Yes, we would agree with this. Printing costs and practicalities had to be a major consideration and this has certainly come at the cost of ergonomics. We have added a comment into the Discussion to discuss this; “The practicality of ensuring printing on a single A4 double-sided sheet of paper does that mean that font size may be challenging for persons with reduced eye-sight, and that the ergonomics of the form may not be optimal.”

- *Was the layout tested in the field to see how best to set out the required information? The paper does not address the following though the authors note that the form “is now in national clinical use in Rwanda"*
- **Response:** The layout was not formally field-tested. But its use has adapted since we have submitted the article for publication. We have therefore added the following text into the clinical implications section “The NRF was not initially field-tested in its double-sided A4 format but has now been subsequently adapted by the Ministry of Health. It has been adapted to fit onto a double-spread pad of carbon-copied paper so that the form can be sent to the receiving hospital, with the sending facility being able to keep a copy of the information being shared.”
- *Does the form work i.e. do clinicians use it reliably where reliability is defined as completion of all required information accurately 100% of the time?*
- **Response:** The form has now been implemented in Rwanda, and its use will make an interesting piece of research work. We have added an additional comment into “Clinical application” to make this a bit clearer.
- *Are any modifications required especially in design of form?*
- **Response**: The initial NRF design was evaluated by Round-4 participants for face validity with modifications applied. Beyond the scope of the “methodology” of this study, the form has now been adapted by the Ministry of Health (as discussed in the point above).
- *Are all the items necessary in real life outside the research environment where it was developed.*
- **Response**: The aimed output was for a NRF suitable for “day-to-day” clinical use, rather than research. We have added this comment into the Objectives and the “Clinical Application” section.
- *What training is required to have the form used? Is there a training manual?*
- **Response**: We have added the following text to the Clinical Implications section – “There is currently no formal training delivered for use of the NRF.”
- *Who fills out the form – nurses or doctors?*
- **Response**: We have added the following text to the Clinical Implications section “In its current use in Rwanda, the NRF is being used at all levels of referral by different professionals. Normal practice is that doctors complete the form in hospitals and nurses and/or midwives complete it at the health center level.”

- *On case by case basis has the form improved the outcome for babies when filled out correctly?*
- **Response**: We aren’t able to comment on this at this point in time in the article. Anecdotally professionals have commented to the authors that the form is helpful in their clinical practice. But this information hasn’t been collected in any formal setting.
- *I note that that these questions are for future research. However a comment on this and on how the implementation of the form is carried out and scaled up would be a good follow up study. A neonatal collaborative across the country may be the way to ensure the form is used on a reliable basis.*
- **Response**: As the Clinical Implications section was getting large we have added a new heading “future research”. We have added the following statement to this section “The design and use of the form have now been taken over by the Neonatal Working Group of the Ministry of Health, which includes multi-disciplinary professionals, who are best placed to evaluate its use.”

**Additional comments from the authors: **Clinical implications section: “The first page also has the potential to be used for intra-hospital transfer.” In the previous version we made this comment. There is a potential for this to be undertaken in Rwanda, but it hasn’t been implemented yet, and we are aware that the section of text is becoming long, therefore we have removed this comment.

# Peer review: Reviewer response for version 1

**DOI:** 10.5256/f1000research.54082.r85767

**Published:** 2021-06-14

**Authors:** Pradyumna Pan

**Affiliations:** 1 Pediatric Surgery Unit, Ashish Hospital and Research Centre, Jabalpur, Madhya Pradesh, India

Well researched and clearly written article. Very informative. In this paper, the authors explored the nature and use of the Delphi research method, explored how and why it may be applied. The authors established the Delphi method being used principally to explore issues and risks as a result of previous research and to forecast future technology application and best practices.

Is the work clearly and accurately presented and does it cite the current literature?

Yes

If applicable, is the statistical analysis and its interpretation appropriate?

Yes

Are all the source data underlying the results available to ensure full reproducibility?

Yes

Is the study design appropriate and is the work technically sound?

Yes

Are the conclusions drawn adequately supported by the results?

Yes

Are sufficient details of methods and analysis provided to allow replication by others?

Yes

Reviewer Expertise:

Surgical outcome of opertion

I confirm that I have read this submission and believe that I have an appropriate level of expertise to confirm that it is of an acceptable scientific standard.
